# The use of toys by nursing as a therapeutic resource in the care of hospitalized children

**DOI:** 10.1590/0034-7167-2022-0433

**Published:** 2023-04-07

**Authors:** Lia Leão Ciuffo, Tania Vignuda de Souza, Thais Mello de Freitas, Juliana Rezende Montenegro Medeiros de Moraes, Keila Cristina Oliveira dos Santos, Roberta de Oliveira Jaime Ferreira Lima dos Santos

**Affiliations:** I Universidade Federal do Rio de Janeiro. Rio de Janeiro, Rio de Janeiro, Brazil; II Universidade Federal do Rio de Janeiro, Instituto de Puericultura e Pediatria Martagão Gesteira. Rio de Janeiro, Rio de Janeiro, Brazil

**Keywords:** Games and Toys, Hospitalized Children, Inpatients Units, Nursing Care, Pediatric Nursing., Juego e Implementos de Juego, Niño Hospitalizado, Unidades de Internación, Atención de Enfermería, Enfermería Pediátrica., Jogos e Brinquedos, Criança Hospitalizada, Unidades de Internação, Cuidados de Enfermagem, Enfermagem Pediátrica.

## Abstract

**Objectives:**

to describe the use of toys by nursing during the care of children in the inpatient unit; to analyze the factors that influence the use of therapeutic toys by nursing in the care of hospitalized children.

**Methods:**

qualitative research, conducted in a pediatric hospital in Rio de Janeiro between July and August 2019. Semi-structured interview and thematic analysis were used as methodological procedure.

**Results:**

the 12 nurses and 7 nursing technicians revealed minimizing fear, relieving tension, and creating a bond between the child and the professional as the main benefits; they use as resources: children’s toys, hospital materials, cartoons, and children’s videos. The high demand for work, deficit of human resources, and appropriate ludic materials are factors that interfere with the use of toys as a therapeutic resource.

**Final Considerations:**

although the participants recognize the importance of the toy as a therapeutic resource, there is no systematization of its use in pediatric practice.

## RESUMO

**Objetivos:**

descrever o uso do brinquedo pela enfermagem durante a assistência à criança na unidade de internação; analisar os fatores que influenciam a utilização do brinquedo terapêutico pela enfermagem no cuidado da criança hospitalizada.

**Métodos:**

pesquisa qualitativa, realizada em um hospital pediátrico do Rio de Janeiro entre julho e agosto de 2019. O procedimento metodológico foi a entrevista semiestruturada e análise temática.

**Resultados:**

os 12 enfermeiros e 7 técnicos de enfermagem revelam como principais benefícios minimizar o medo, aliviar a tensão e criar vínculo entre criança e profissional e; utilizam como recursos: brinquedos da criança, materiais hospitalares, desenhos animados e vídeos infantis. A alta demanda de trabalho e o déficit de recursos humanos e materiais lúdicos apropriados são fatores que interferem no uso do brinquedo como recurso terapêutico.

**Considerações Finais:**

embora os participantes reconheçam a importância do brinquedo enquanto recurso terapêutico, não há sistematização de sua utilização na prática pediátrica.

## RESUMEN

**Objetivos:**

describir uso del juguete por la enfermería durante la atención al niño en unidad de internación; analizar los factores que influencian la utilización del juguete terapéutico por la enfermería en la atención al niño hospitalizado.

**Métodos:**

investigación cualitativa, realizada en un hospital pediátrico de Rio de Janeiro entre julio y agosto de 2019. Procedimiento metodológico fue la entrevista semiestructurada y análisis temático.

**Resultados:**

los 12 enfermeros y 7 técnicos de enfermería revelan como principales beneficios minimizar miedo, aliviar tensión y construir vínculo entre niño y profesional; utilizan como recursos: juguetes del niño, materiales hospitalarios, dibujos animados y videos infantiles. La alta demanda de trabajo y el déficit de recursos humanos y materiales lúdicos apropiados son factores que interferen en el uso del juguete como recurso terapéutico.

**Consideraciones Finales:**

aunque los participantes reconozcan la importancia del juguete mientras recurso terapéutico, no hay sistematización de su utilización en la práctica pediátrica.

## INTRODUCTION

The process of hospitalization is a difficult confrontation for children and their families. The child’s routine undergoes changes, and the stay in the hospital environment triggers stress, fear, anxiety, and often, emotional trauma. In this sense, such a situation may compromise their physical and psychological health, in addition to hindering the ability to cope with procedures, causing changes in behavior and impairing recovery^(1-4)^.

In pediatric hospital practice, it is common to have toys available in the inpatient unit or in the playroom, where children play freely or under the responsibility of a pedagogy professional. However, toys can and should be used by the nursing team to facilitate interaction, communication, and the expression of feelings^(2-3)^, as well as to help in facing the hospitalization process and in preparing for diagnostic and therapeutic procedures^(5-7)^.

To offer the child the opportunity to use toys to simulate the procedures performed allows for the clearing of possible doubts, as well as minimizes fear and insecurity and facilitates the understanding of what will be performed^(1)^.

According to the first article of Resolution No. 546/2017 of the Federal Nursing Council, it is up to the nursing team working in the pediatric area to use the toy technique/therapeutic toy (TT) in the care of hospitalized children and families. The sole paragraph recommends that this technique, when used by the nursing technician, should be prescribed and supervised by the nurse^(8)^.

A study developed in a maternal and child hospital revealed that the main reason reported by nurses for not routinely including toys in their childcare actions was related to a reduction of human and material resources and to the nursing staff work routine, which is usually divided into bureaucratic-administrative and care actions^(5)^.

In a study carried out in pediatric inpatient units of a university hospital in the city of São Paulo, it was revealed that, although nurses recognize the various benefits of the use of toys/TT, their use is unsystematized and heterogeneous; therefore, training of these professionals and effective implementation is needed to systematize their practice^(6)^.

Given the importance of the topic (nurses’ competence in the use of toys/TT), the object of the study was: the use of toys by the nursing team as a therapeutic resource in a hospital inpatient unit.

## OBJECTIVES

To describe the use of toys/TT by the nursing team during the care of children as a therapeutic process; to analyze the factors that influence the use of therapeutic toys by nursing in the care of hospitalized children.

## METHODS

### Ethical aspects

Data collection occurred after the approval by the Research Ethics Committee of the participating institution. Once the project was approved, the participants were consulted, informed about the research objectives, and invited to participate; afterwards, they voluntarily signed the Informed Consent Form (ICF). The study complied with Resolutions No. 466/2012 and No. 510/2016 of the National Health Council.

### Study Type

Qualitative research, which is based on the subjective aspect and on the relationships within the reality in which the social actors are inserted, searching for meanings, motives, values, and beliefs^(9)^. By adopting this type of approach, it is possible to capture the meaning of the implementation of the toy/therapeutic toy in the nursing care provided to hospitalized children. The COREQ (Consolidated criteria for reporting qualitative research) instrument was used.

### Methodological procedures

The semi-structured interview technique^(10)^ was used, which allows other questions to be added, aiming at deepening answers, enabling a more dynamic dialog. The interview script consisted of the following questions: What is your experience in using the toy/TT during child care? What strategies or dynamics do you use in the absence of the toy/TT? What facilities and difficulties have you encountered using the toy/TT during nursing care?

### Study setting

The study setting was the inpatient unit of a pediatric hospital located in the city of Rio de Janeiro. The unit is composed of six pediatric wards divided by age groups and contains 46 beds.

The choice of this scenario is justified because it is an inpatient sector for children of different age groups (29 days to 13 incomplete years) and with several clinical and surgical diagnoses that require nursing care, in addition to painful and invasive procedures.

### Data source

Nurses and nursing technicians working in the pediatric inpatient unit participated in the study. All those invited participated voluntarily and worked on the day or night shift. There was no refusal to participate or request to withdraw from the study after data collection. Inclusion criteria were nursing professionals who had worked in the sector for at least six months and were available at the time of collection. Nursing professionals who worked in the sector temporarily to cover vacations or leaves of other professionals were excluded.

To maintain the participants’ anonymity, they were identified with codes formed by the initials of their profession (NUR for nurses and TEC for nursing technicians) followed by numbers, indicating the order of the interviews. Each interview lasted an average of ten minutes.

Five steps were used for theoretical saturation^(11)^: 1 - recording of raw data (primary sources: transcription of the interviews); 2 - data immersion (skimming through literature); 3 - compilation of each interview’s individual analyses and thematic grouping (organization of the data by colorimetric coding); 4 - allocation of the themes and types of statements in a thematic framework (identification of the regularity of the findings in the statements according to the themes, and verification of the consistency of the statements); and 5 - verification of the theoretical saturation of the data by identifying the absence of new elements in each thematic grouping.

### Data collection and organization

Data collection occurred in July and August 2019. Prior the interview, the researchers read the ICF to the participants, outlining the objectives of the study and inviting them to participate. After acceptance, the participants signed and initialed the ICF; afterwards, a copy of the document was given to the professional. At that moment the researchers scheduled the day and time of the interviews, which took place in a previously reserved room, with the objective of allowing audio recordings (in a digital file) without noise and/or interference from the inpatient unit. After each interview, a transcription of the recordings was done in a Microsoft Word software document. Afterwards, the participant was contacted to read the material and verify the veracity of the data provided or indicate the need for alteration, thus ensuring the internal validity of the data.

### Analysis of results

Data analysis was based on thematic analysis^(9)^ and was carried out in the following stages: “pre-analysis”, which consists of skimming and thorough reading of the material, formulation and reformulation of guiding questions, and meeting the objectives; “material exploration”, which aims to understand the analyzed text and organize the content into theoretical or empirical categories; and the third and final stage, called “treatment and interpretation of data”, in which the researcher makes inferences and interpretations about the results.

Thus, the categories were named “The toy and its benefits: its use by the nursing team as a therapeutic resource”; and “Factors that interfere with the implementation of the toy/therapeutic toy”.

## RESULTS

The sample included 12 nurses and 7 nursing technicians.

### The toy and its benefits: its use by the nursing team as a therapeutic resource

This topic addresses the benefits of using toys during nursing care in the pediatric inpatient unit, as well as their use as a therapeutic resource.

Of the 19 research participants, 7 highlighted that the toy provides several benefits during the child’s hospitalization, among them: it minimizes feelings of sadness or brings joy; aids in the child’s recovery; facilitates interaction with the professional; minimizes pain and discomfort during the procedure; causes sensory stimuli; makes the child calmer and less stressed, facilitating acceptance of the procedure; allows for the creation of bonding and trust in the professional:

[...] *it’s essential for those who work in pediatrics because the children cheer up and are no longer sad because of this method* [...]. (TEC1)

*I think that the toy helps in the child’s recovery* [...] *the child* [...] *is more accepting as well* [...]. (TEC 3)

[...] *it is visible and notorious the effectiveness of playing during the procedure; many times, we are able to win the child over with playing* [...]. (NUR 2)

*Well, me, personally, use it with the intention of trying to minimize the child’s pain and discomfort during any given procedure.* (NUR 8)

[...] *the use of toys interferes in changing the child’s behavior; and today we have a wide variety of toys that really stimulate the child: they are colorful, stimulate the numerical side and sensorial stimulation. So, we can see how valuable this is in our day to day.* (TEC 7)

[...] *makes the child calmer, more tranquil, thus reducing the stress generated by this procedure* [...] *so this helps a lot in creating a bond between the child and the professional, and the child ends up having more confidence when we approach what is normal for him, which is playing, the ludic.* (NUR 11)

[...] *it is a way to try and get the child to calm down and feel a bit safer.* (NUR 12)

It is noted that some participants make comments about objects and features of clothing and use the resources found in the child’s unit, such as a lipstick, a doll, procedure gloves or medical devices, a book, and cartoons and children’s videos, according to the following statements:

[...] *I try to play, talk about the lipstick, the clothing, I don’t know, I try to interact more* [...]. (NUR 1)

[...] *usually, she has a doll close by, so I take the doll and have her perform the dressing on the doll first* [...] *then I do it on her* [...]. (NUR2)

[...] *during* [...] *a peripheral venipuncture, he didn’t even want to take the toy, so* [...] *I used a video with children’s music in which he calmed down* [...]. (NUR 3)

[...] *sometimes I use this dynamic of playing with the doll* [...]. (NUR 4)

[...] *we use the instruments we use daily at work to make toys, like gloves* [...] *we use children’s videos* [...]. (NUR 5)

[...] *in some situations, for example, before surgical procedures, where the child has a longer waiting period, I try to interact with a book or use the playroom so that the child can play while waiting.* (NUR 9)

*I* [...] *show her the materials I am going to use and, if possible, let her touch them to see what they look like* [...]. (NUR 12)

[...] *a cartoon* [...] *helps a lot because she gets distracted, she doesn’t even notice what you are doing.* (TEC 2)

[...] *here, sometimes, we put on a cartoon for the child to watch, sing a song, things like that.* (TEC 4)

In the statement of nursing technician 5, it is evident that entertaining or commenting on something that can entertain the child is at the discretion of each professional:

[...] *whenever possible we try to be playful, to be doing something for the children to have fun, but that’s up to us, understand?* (TEC 5)

### Factors that interfere with the implementation of the toy/therapeutic toy

This topic addresses the factors that interfere with the implementation of the toy as a therapeutic resource, such as: high work demand; deficit of material and human resources; and inappropriate environment.

Out of the 19 interviewees, 3 stated that the demands of work and/or intercurrences, routine procedures, and mechanical care, as well as the lack of time due to the work process, contribute to professionals not valuing the use of the toy as a resource:

[...] *I think that the work demand ends up getting in the way, because sometimes it is a lot, then, when things are very bad, a day of many procedures, of many intercurrences, I find it to be even unfeasible, you know?* (NUR 4)

[...] *you end up getting into the daily routine, of the procedures, the time to have to deal with everything, to do everything, then we end up leaving it aside* [...]. (NUR 6)

[...] *sometimes, we become very mechanistic, doing the procedure in a very mechanical way, for example: one goes in, punctures, then goes out, then another one goes in, and sometimes we end up omitting these strategies* [...]. (NUR 7)

[...] *the day is always very busy, with many tasks* [...]. (NUR 9)

Another difficulty revealed is the lack of material resources for the use of the TT during technical procedures, leading nursing professionals to use their personal objects:

*I think the difficulty is the fact that we don’t have any objects for collective use. It would be good to create these objects, because sometimes we do things that we aren’t allowed, like using our personal objects to entertain* [...]. (TEC 5)

The shortage of human resources was also cited as something that hinders the use of the toy/TT:

[...] *the scarcity of professionals in the workplace, because this affects the progress of the service a lot* [...]. (TEC 6)

Nurse 5 justifies the use of work tools as she doesn’t have access to the playroom (physical space for games and recreation).

[...] *because we don’t have much access to the playroom, so it makes it difficult for us to use the toys.* (NUR 5)

Nurse 10 reports that there was training in the hematology unit on the use of dolls as a TT; however, when trying to implement it, it failed for two reasons: the child treated in hematology goes through various sectors, in which it was not possible to train professionals to use the doll; and the child did not carry the doll with her when moving between sectors:

[...] *there was a period when they decided to implement the therapeutic toy here in the institution with the children in hematology* [...]. *In this training, we realized that here the environment is not propitious because the child in hematology goes through several different sectors, such as the blood bank, the “aquário carioca”* [sea-themed room for children undergoing chemotherapy]*, the infirmary, the outpatient clinic, and the emergency room, and not everyone received the doll training, for this reason I think it was not implemented. We did receive some dolls, some people even implemented it, but the children were not bringing the doll, so the doll itself ended up being of very little value.* (NUR 10)

On the other hand, according to the statements of two nurses, besides not having any experience with the use of toys/TT, they don’t recognize this use in the studied scenario:

*Here* [...] *I haven’t had much of this therapeutic toy experience and I don’t even see it being used.* (NUR 7)

[...] *I never saw the therapeutic toy where I work* [...] *I know that it is used, that some institutions use the therapeutic toy* [...] *that it is a toy assembled for a certain procedure, which is directed to a certain case, but I don’t use it* [...]. (NUR 12)

## DISCUSSION

For hospitalized children, the hospitalization process can become a traumatizing experience since they undergo several changes in their daily lives, such as the family environment, school, and friends; they are submitted to some restrictions, several invasive, uncomfortable, and painful procedures; and are confronted with new experiences that generate feelings of different orders, such as fear, anger, insecurity, and uncertainty^(1,12-13)^. Moreover, it is necessary to consider that there is limited contact with other children and that their interaction with adults during play does not occur in the same way as when they are with their peers^(14)^.

One of the play modalities often used by the nursing team is the TT, which, besides distracting, helps the child to accept care, especially regarding experiences that usually cause fear and threat^(1,13)^. It also helps to reduce anxiety arising from threatening and atypical situations, helping the child to face changes in routine. In addition, it can be implemented to evaluate the child’s understanding of experiences in an environment different from his/her usual one and whenever he/she needs to understand and deal with daily events^(1-2)^.

The TT has a specific purpose and should be incorporated during nursing care to establish communication and relationship with the child, to know his/her feelings and concerns, to help relieve his/her tension and anxiety, and to prepare him/her for procedures^(1-2,15)^. Thus, the use of play in a hospital environment is a catalyst in the child’s adaptation process because playing is an appropriate strategy for coping with hospitalization^(5-6)^.

Corroborating this line of thought, studies highlight that humanized actions in nursing care through playful resources collaborate to strengthen the bond between nursing professionals and children, facilitating communication and promoting an interaction that minimizes possible fears of the child, because they promote moments of distraction and relaxation^(3,5,16)^. Furthermore, play, when inserted into the context of treatment for example, can confer precious collaboration to the health team^(13,17-19)^.

Among the benefits of play in child care, the minimization of the effects of hospitalization and the stimulation in the adaptation process stand out, because playing seeks to achieve joy, relaxation, and the shaping of a more pleasant environment; this favors the interaction between the professional, the child, and his/her family, as well as, even if momentarily, takes the focus off the disease, helping in the adaptation and coping with the health-illness process and hospitalization^(1-2,5,7)^. Thus, its therapeutic value is revealed, contributing to the physical and emotional well-being and recovery of hospitalized children^(17-20)^.

According to a study, children who received ludic interventions in the hospital during their hospitalization had fewer negative emotions, as well as lower levels of anxiety compared to those who received usual care^(5)^.

It was observed that the nursing professional, even when facing difficulties, tries to create conditions for better interactions with the child, commenting on the characteristics of the clothes she uses or making use of medical devices that she will use in the procedure, dolls that are in the child’s unit, children’s videos, cartoons, and books.

If on the one hand the participants refer that they have no experience with the TT and don’t see it being used in practice, on the other, some nursing professionals manage to use the resources made available in the children’s unit. According to some statements, the TT is an object built specifically for a certain technical procedure. However, for others, it can be something improvised, and it is important for these professionals to interact with the child to minimize the fear of pain and maintain confidence in the professional, reducing stress, among other problems; therefore, the toy is considered a therapeutic resource.

The study participants showed that they had theoretical knowledge about the TT and used a few existing resources in the unit, the setting of the study, due to the lack of a specific toy for this purpose; high work demands and/or intercurrences; lack of time to develop all the activities; and a deficit in human resources that lead to a mechanistic practice and lack of appreciation of the TT.

Indeed, in Brazil, the nurse is responsible for many bureaucratic and care activities; however, the need to perceive play as a therapeutic resource as important as the performance of a technical procedure is reinforced. Therefore, in their activities of organizing care in their unit, the nurse should foresee and plan how to insert play in the care plan of each child^(3)^.

In this sense, play should be made available in different situations (physical examination, nursing procedures, therapeutic communication) and hospital spaces (bed, playroom, procedure room). It is also important that different types of toys or play strategies (tone of voice, use of colored stethoscopes, toys adapted with health devices) be available according to age, and that infection prevention actions are performed so that the toys offered to children are safe^(3)^. So, the nursing team must know how to use them as a communication and interaction strategy in the assistance to minimize possible traumatic effects on children caused by hospitalization^(16-17)^.

To this end, it is necessary to consolidate the practice of playing with the teams working in pediatric units due to the beneficial effects of playing, which favorably impact the child’s confidence and mood, as well as strengthen their relationships with health professionals, family, and themselves, favoring the healing process^(16,21)^.Thus, promoting and implementing approaches that provide care through play has great value, as they allow for the approximation, the communication process, and greater interaction of the nursing team with the child and his family^(17,21)^.

### Study limitations

Since it is qualitative, the approach was restricted to only one public pediatric institution, which does not allow for the generalization of the results. Therefore, other studies are recommended, including those that demonstrate the participation of the nursing team in the private setting.

### Contributions to the field of Nursing

The study contributes to nursing care in the sense of stimulating the reflection of professionals on the use of toys, objects, and clothing of the child itself, medical devices, and children’s videos and cartoons in the perspective of a playful and welcoming approach, using them as a therapeutic resource. It will also be a secondary source of research on the subject. Furthermore, the study stimulates the development of new research on the subject in other settings.

## FINAL CONSIDERATIONS

The nursing professionals participating in this study revealed they use some toys made available in the child’s bed, as well as hospital materials, and cartoons and children’s videos, in addition to making comments about characteristics of the child’s clothing. However, there is no standardization of the technique, meaning that this is at the discretion of each professional. These activities are performed with the purpose of distracting the child and making him/her accept more easily the technical procedure that will be developed; and, also, to provide some benefits such as: minimizing fear, relieving tension, and promoting the creation of a bond between the child and the professional. However, the high demand for work and the deficit of human and material resources are some factors that end up interfering with the implementation of such resources.

On the one hand, the nursing professional understands the use of the TT as a strategy to minimize the fear of pain, to get closer to the child, and for the child to trust him/her. On the other hand, the high workload and the little availability of these resources make its implementation difficult.
